# Phenotypic Diversity Analysis in the Sect. *Tuberculate* (*Camellia* L.) Population, an Endemic Taxon in China

**DOI:** 10.3390/plants13223210

**Published:** 2024-11-15

**Authors:** Zhaohui Ran, Xu Xiao, Lei Zhou, Chao Yan, Xinxiang Bai, Jing Ou, Zhi Li

**Affiliations:** College of Forestry, Guizhou University, Guiyang 550025, China; ranzhaohui1998@outlook.com (Z.R.); xiaoxu199801@163.com (X.X.); 15085594016@163.com (L.Z.); godblessuuus@126.com (C.Y.); xxbai@gzu.edu.cn (X.B.)

**Keywords:** sect. *Tuberculate*, phenotypic trait key, cluster analysis, phenotypic diversity

## Abstract

Sect. *Tuberculate* Chang belongs to the genus *Camellia*, which is an endemic group in China and has high research value. However, the phenotypic patterns of this taxon are complex and diverse, and the phenotypic variation in key traits is still unclear. In this study, a total of 212 samples from 18 populations of sect. *Tuberculate* plants were studied for 30 phenotypic traits of flowers, fruits, and leaves using analysis of variance, correlation analysis, principal component analysis, and cluster analysis. The results showed the following. (1) The plants in sect. *Tuberculate* were rich in phenotypic trait variation and possessed rich phenotypic diversity. The differentiation of phenotypic traits mainly came from among populations, with leaves (66.804%) being the largest and flowers (53.476%) being the smallest. Qualitative traits (70.264%) were greater than quantitative traits (57.608%). (2) Correlation analyses showed close and complex relationships among the phenotypic traits of flowers, fruits, and leaves. (3) The cumulative contribution of the first 10 principal components was up to 73.49%, which screened out 12 major traits contributing to the phenotypic differences in plants of sect. *Tuberculate*. (4) Q-type analysis showed that they were classified into 18 taxa at a Euclidean distance of 7.5 and 11 taxa at a Euclidean distance of 10. The 18 populations were not fully clustered according to the geographic distance of the plants, and there was an overlap between some of the populations. In summary, the degree of variation in phenotypic traits among populations of sect. *Tuberculate* plants is high, which is affected by the climatic environment. The 12 major phenotypic traits screened can be used as the basis for the classification of sect. *Tuberculate* plants. There are trait overlaps among some populations, which may be affected by the stochastic influence of the geographic climate and gene flow. This study will provide important references for interspecific identification, classification system construction, genetic mechanism, germplasm resource conservation, and exploitation of plants in sect. *Tuberculate*.

## 1. Introduction

Plant phenotypic traits are measurable physical, physiological, and biochemical characteristics exhibited by plants during growth and development, and these traits directly reflect the composition and structure of plants and their growth and development processes [1,2]. Phenotypic traits are an important bridge between genotype and environment, and phenotypic variation usually occurs in plants after long-term natural selection and environmental stress, as it is a survival strategy for plants to adapt to different habitats [3]. The observation and analysis of phenotypic traits can reveal the effects of genotype–environment interactions on plant growth and development, providing an important basis for genetic breeding. For example, in crop breeding, new varieties with excellent traits can be bred through phenotypic selection [4]. At the same time, phenotypic traits are also a direct reflection of a plant’s adaptation to the environment. Plants adapt to different environmental conditions, such as drought, salinity, pests and diseases, and other adversities by adjusting their morphological structure, physiological mechanisms, and other phenotypic traits, and such adaptation is the key to the survival and reproduction of plants [5,6,7]. Morphological classification is the most important tool for phenotypic studies, and it is a simple, intuitive, and visual way to identify plant species based on differences or similarities in their external morphology [8,9]. Therefore, based on the results of phenotypic traits, a reasonable plant classification system can be constructed to provide a framework and guidance for botanical research, and it is the initial way to select and breed good plant varieties, which is of great significance for the conservation and utilization of rare plants.

The leaf is the main organ for photosynthesis in plants, and its morphological characteristics directly affect the efficiency of photosynthesis and transpiration in plants [10]. The morphological features of flowers and fruits, the reproductive organs of plants, are important for insect pollination and seed dispersal [11]. For example, plants with large and broad leaves capture more sunlight, which improves the efficiency of photosynthesis and provides more energy and nutrients to the plant. Such leaves also help the plant to carry out transpiration and regulate body temperature and water balance [12]. In recent years, the phenotypic traits of flowers, fruits, and leaves have been widely used in the study of the phenotypic diversity of the genus *Camellia* to reveal the degree of variation in phenotypic traits and their diversity characteristics [13,14,15].

The plants in the sect. *Tuberculate* Chang Tax. belong to the genus *Camellia* in the family Camelliaceae. They are named for their “verrucose protuberances on the surface of the ovary and fruit” and are considered to be one of the genera of *Camellia* that have maintained their original shape [16]. Plants of this group are mostly shrubs or small trees, with thin leathery or leathery leaves of various shapes, such as oblong, lanceolate, or obovate. The flowers are solitary or in pairs, white, sessile, or short-stalked, and the fruits are mostly capsules, with verrucose bumps on their surfaces [16,17,18,19,20,21]. The number of known plant species in sect. *Tuberculate* has varied, with Chang et al. suggesting 18 species [19,20] and Min et al. suggesting 6 species and four variants [16]. This taxon is concentrated in the southwestern region of China, especially centered in Guizhou and spreading to neighboring provinces [22]. Guizhou Province, with its complex and diverse types of topography and climate, is one of the most typical regions in the world for the development of karst landforms [23]. Its unique geographic location and climatic conditions provide favorable conditions for harboring rich biodiversity, while the vegetation types and species distribution in the region show strong differences. It was found that some species of sect. *Tuberculate* plants, such as *Camellia rhytidophylla* (ZYL) and *Camellia rubimuricata* (LBL), have higher light energy conversion efficiency and stronger heat dissipation ability and are better adapted to grow in the field under natural light irradiation, whereas some other species, such as *Camellia neriifolia* (XYL) and *Camellia parvimuricata* (XLG), are sensitive to light, as they are especially poorly adapted to growing in the field under strong light irradiation in the summer [24]. *Camellia rubituberculata* (HKH) has high ornamental value because of its elegant plant shape, elegant flowers, and peculiar fruit shape, and it can be used as an ornamental plant in a garden or for landscaping [25]. The fruit contains more trace elements and has good medicinal value. The wood is hard and delicate, making it an excellent timber for agricultural tools and joinery. At the same time, the fruits and seeds may also have potential economic value, such as for oil extraction or as other industrial raw materials [26]. In summary, plants of sect. *Tuberculate* have important applications in ornamental, ecological, economic, and scientific research.

As an endemic taxon in China, the study of sect. *Tuberculate* plants mainly focuses on the classification of species, leaf anatomy, chloroplast genome, seed germination, and species distribution pattern [26,27,28,29,30,31]. Few studies on their phenotypic traits have been reported, and there is a lack of studies on the correlations between the flowers, fruits, and leaves of sect. *Tuberculate* plants and environmental factors, greatly hampering the development and utilization of their resources. Some of the species in this group have similar morphological characteristics and are often easily confused, so it is important to accurately differentiate their species groups. In the study, 212 samples from 18 populations of sect. *Tuberculate* plants were selected for the investigation and measurement of the phenotypic traits of flowers, fruits, and leaves. Our main objectives were (1) to analyze the diversity and the degree of variation among different populations and individuals, (2) to clarify the links between phenotypic traits and environmental factors, and (3) to identify the main taxonomic traits among populations and their geographic distribution characteristics. The goal was to provide a scientific basis for species identification, conservation of germplasm resources, genetic improvement, and development and utilization of sect. *Tuberculate* plants.

## 2. Materials and Methods

### 2.1. Collection of Plant Material

**Figure 1:**
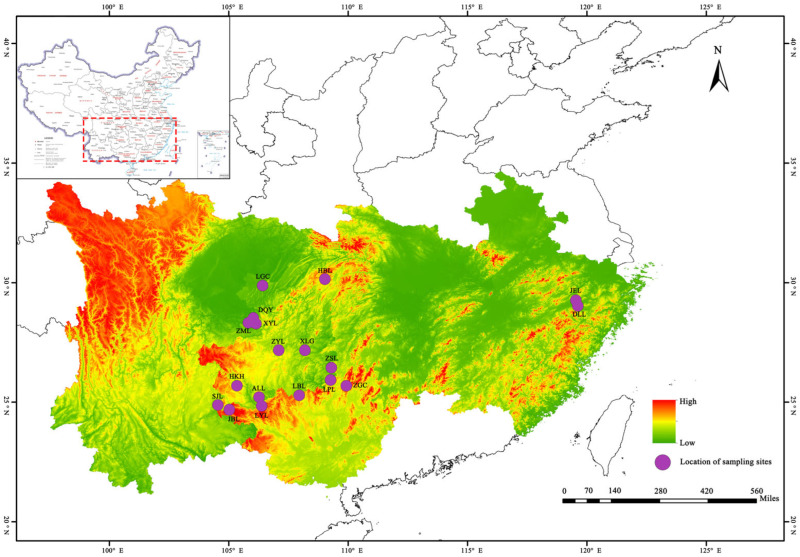
Geographical distribution of different groups of sect. *Tuberculate* plants. (ALL: *Camellia anlungensis* Chang; DLL: *Camellia obovatifolia* Chang; DQY: *Camellia ilicifolia* Y.K.Li; HBL: *Camellia hupehensis* Chang; JBL: *Camellia acutiperulata* Chang and Ye; JEL: *Camellia acutisepala* Chang; LBL: *Camellia rubimuricata* Chang and Z.R.Xu; LGC: *Camellia tuberculata* Chien; LPL: *Camellia lipingensis* Chang; LYL: *Camellia leyeensis* Chang and Y.C.Zhong; HKH: *Camellia rubituberculata* Chang and Yu; SJL: *Camellia pyxidiacea* Xu F.P.Chen and C.Y.Deng; XLG: *Camellia parvimuricata* Chang; XYL: *Camellia neriifolia* Hung T. Chang; ZGC: *Camellia rhytidocarpa* Chang and Liang; ZML: *Camellia atuberculata* Chang; ZSL: *Camellia zengii* Chang; ZYL: *Camellia rhytidophylla* Y.K.Li and M.Z.Yang).

The distribution range of the plants in sect. *Tuberculate* was determined based on an extensive review of the literature, specimen checking, and a prior field investigation. Then, 212 healthy adult plants from 18 populations of sect. *Tuberculate* were selected for the study according to their distribution ranges (Table 1). Most of this taxon grows at moderate elevations in mountainous areas, along river valleys, or in evergreen forests in moist, well-drained soil environments. As a result, populations of some species are small, and sampling is difficult. In sampling, 2–17 healthy, growing adult plants were selected per population (in principle, more than 10 plants were selected per population), and the distance between samples was greater than 20 m, thus reducing the kinship between them. The Global Positioning System (GPS) location was utilized to record the geographic location information of each sample, as shown in Figure 1. Meteorological data from 1991 to 2020 were downloaded from the China Meteorological Administration (https://weather.cma.cn, accessed on 7 September 2024), and the details are shown in Table 1.

### 2.2. Determination of Phenotypic Traits

Based on the descriptions and identifying features of the species of sect. *Tuberculate* in *Flora of China* [21], as well as observations of herbarium and field samples, flowers, fruits, and leaves of the plants in sect. *Tuberculate* were selected for measurements. In each of these plants, 10 groups of the upper, middle, and lower parts of leaves were selected, 10 fruits were selected, and 10 flowers were selected using a typical, standardized, and consistent selection of blooming flowers from the middle and upper parts of the division. Finally, the mean values were calculated for statistical analysis. Eventually, data on 30 phenotypic traits were obtained from 212 plants of sect. *Tuberculate*, including 9 qualitative traits (Table 2) (type of leaf (TL), leaf shape (LS), leaf bases (LB), leaf color (LC), leaf margin (LM), leaf apex (LEA), type of splitting fruit (TF), flower color (FC), and calyx apex (CA)) and 21 quantitative traits (Table 3) (leaf length (LL), leaf width (LW), petiole length (PEL), leaf area (LA), leaf shape index (LSI), leaf veins (LV), fruit diameter (FD), fruit stalk length (FSI), fruit length (FL), fruit shell thickness (FST), seed length (SDL), number of ovaries (NO), number of seeds (NS), number of calyxes (NC), calyx length (CL), calyx width (CW), number of petals (NP), petal length (PL), petal width (PW), style length (STL), and number of styles (NOS)). The data on the plant quality traits of sect. *Tuberculate* were graded and standardized with reference to the specification for the description of tea germplasm resources and data standards [32,33].

**Figure 2:**
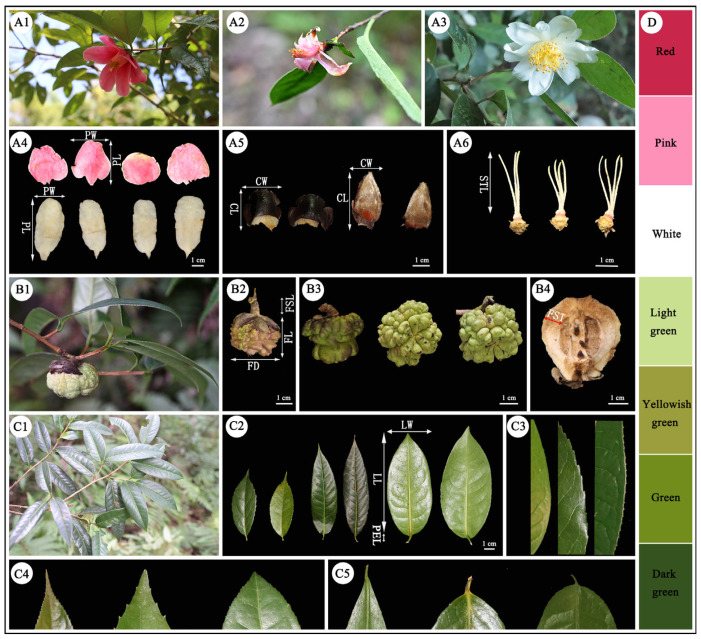
Phenotypic traits of plants of sect. *Tuberculate*. ((**A1**–**A3**): Flowers color and morphological characteristics; (**A4**): Measurement of petals; (**A5**): Measurement of the calyx; (**A6**): Measurement of styles; (**B1**): Morphological characteristics of the fruit; (**B2**): Morphological measurements of the fruit; (**B3**): Types of splitting fruits; (**B4**): Measurement of fruit shell thickness; (**C1**): Morphological characteristics of the leaf; (**C2**): Size and measurements of the leaf; (**C3**): Leaf margin; (**C4**): Types of apices; (**C5**): Types of bases; (**D**): Color criteria of flowers and leaves).

The specific measurements of quantitative traits were as follows (Figure 2). (1) Flowers: The petal length (length from petal base to top), petal width (length at the widest part of the petal), calyx length (length from sepal base to top), calyx width (length at the widest part of the sepal), and style length (length from the base of the style to the stigma) were measured using a vernier caliper, and the number of calyxes, number of petals, and number of styles were counted. Six replications were performed for each trait, and the results were averaged. (2) Fruit: Vernier calipers were used to measure the fruit length (length of the longest axis of the fruit), fruit diameter (width of the fruit at its widest point), fruit stalk length (length of the fruit from the base of the fruit to the end of the stalk), fruit shell thickness (the average of the thickest part of the fruit’s husk, the thinnest part of the husk, and the middle part of the fruit), and seed length, as well as to calculate the number of ovaries and the number of seeds. Six replications were performed for each trait, and the results were averaged. (3) Leaf: Using vernier calipers, the leaf length (length of the leaf blade from base to tip), leaf width (length of the leaf blade at its widest point), leaf area (projected area of leaf blade from the base to top), and petiole length (base of the leaf blade to where the petiole met the stem) were measured, and the leaf veins (number of pairs of veins between the main and lateral veins of the leaf blade) and the leaf shape index (the ratio of the leaf length to the leaf width) were calculated. Six replications were performed for each trait, and the results were averaged.

### 2.3. Data Analysis

The mean, standard deviation (SD), coefficient of variation, and Shannon–Weaver genetic diversity index (H) of the measured phenotypic trait data were assessed using Microsoft Excel 2021 software. ArcGIS 10.6 software was used to produce a geographic distribution map of the populations. The data on phenotypic traits were standardized using SPSS 19.0 software for multiple comparisons, nested ANOVA [34], correlation analysis using intergroup linkage with Pearson’s correlation coefficient as a metric, R-type cluster analysis, and Q-type cluster analysis using Ward’s method with the squared Euclidean distance as the genetic distance [35]. Principal component analysis (PCA) graphs were analyzed using Origin 2021 software.

The coefficient of variation (CV), phenotypic differentiation coefficient (Vst), and Shannon–Wiener diversity index (H) were used as direct measures of genetic diversity and the degree of trait variation. CV denotes the degree of trait dispersion and is calculated as CV/% = s/x × 100, where x is the trait mean and s is the standard deviation [36]. Vst represents the percentage of among population variance to the total variance of the trait and is calculated as Vst/% = [δ^2^t/s/(δ^2^t/s + δ^2^s)] × 100, where δ^2^t/s is the among population variance component and δ^2^s is the within population variance component [37]. The Shannon–Weaver genetic diversity index is calculated as H = −∑Pi × lnPi, with Pi being the percentage of the number of copies of material within level i of a trait with respect to the total number of copies [38,39]. In the quantitative trait data, all of the samples were divided into 10 levels. In the quantitative trait data, all samples were classified into 10 levels, from level 1 [Xi < (X − 2s)] to level 10 [Xi > (X + 2s)], with one level every 0.5s. Xi were the data for level i. The relative frequency of each level (Pi) was used to compute the Shannon–Weiner diversity index (H).

## 3. Results

### 3.1. Differentiation of Phenotypic Traits Among and Within Populations of Sect. Tuberculate Plants

The variance components and phenotypic differentiation coefficients for 30 phenotypic traits among and within populations of sect. *Tuberculate* plants were calculated using the nested variance method. The results showed that the phenotypic traits of flowers, fruits, and leaves of sect. *Tuberculate* differed considerably among populations. The variance components of the 30 phenotypic traits among populations and within populations were 41.152–50.661% and 3.483–50.266%, respectively, and the means of the variance components were 47.363% and 32.705%, indicating that the differentiation of phenotypic traits was mainly among populations (Table 4). There were 19 phenotypic traits that were significantly different among populations and 11 phenotypic traits that were significantly different within populations, indicating that most of the traits were highly variable among populations and not significantly different within populations. The mean value of the phenotypic differentiation coefficient (Vst) for the 30 phenotypic traits was 61.405%, with a minimum FC of 45.026% and a maximum LC of 93.214%. Overall, the mean values of the phenotypic differentiation coefficients of plants in sect. *Tuberculate* showed that flowers (53.476%) < fruits (64.209%) < leaves (66.804%) and qualitative traits (70.264%) > quantitative traits (57.608%).

### 3.2. Analysis of Variance for Phenotypic Traits

As shown in Table S1, by analyzing the variance of 21 quantitative traits in 18 populations of sect. *Tuberculate* with the mean, standard deviation, and coefficient of variation, the mean coefficient of variation was 14.88%. The results showed that there was abundant variation in the phenotypic traits of plants in sect. *Tuberculate*. The average coefficient of variation among different populations ranged from 10.05% to 19.33%, with *Camellia pyxidiacea* (SJL) being the highest and *Camellia rhytidocarpa* (ZGC) being the smallest, and there were nine populations for which it was greater than 15%. The SJL population had the richest degree of variation in phenotypic traits. The coefficient of variation among the different traits ranged from 6.66% to 27.98%, with FSL being the largest. It was also high for LA (26.96%), PEL (23.56%), FST (22.59%), and SDL (20.91%), indicating rich variation. The coefficient of variation for NP (6.66%) was smaller, and it was relatively stable. The mean values of the coefficient of variation showed the order fruit (18.57%) > leaf (17.85%) > flower (9.41%).

### 3.3. Diversity Evaluation of Phenotypic Traits

The 21 quantitative traits of sect. *Tuberculate* plants were analyzed, and the results showed (Table S1) that the Shannon–Weaver genetic diversity indices for quantitative traits ranged from 0.76 to 2.07, with a mean value of 1.72. Of these, 18 traits had Shannon–Weaver genetic diversity indices greater than 1, with the smallest being for NO (0.76) and the largest being for LL and PEL (2.07). The nine quality traits of sect. *Tuberculate* plants showed a total of 29 variant types with high diversity (Table 5). Three quality traits had Shannon–Weaver genetic diversity indices greater than 1. TL (1.27) had the largest and LM (0.48) had the smallest, with a mean value of 0.88. The characterization of the traits showed that medium-sized leaves accounted for 43.87% of the total, lanceolate leaves accounted for 50.94% of the total, leaf bases were predominantly wedge-shaped (63.21%), and leaf blade color types were more varied and predominantly green (35.85%) and dark green (29.25%). Leaf margins were mostly undulate (85.85%). The type of acuminate leaf apices accounted for 48.58% of the total, vertical fracture as the type of fruit cleavage (81.60%) accounted for 81.60% of the total, white flowers accounted for 90.57% of the total, and an apex as the calyx type (51.89%) and rounded calyx type (48.11%) were present in small proportions. Overall, the phenotypic diversity index for quantitative traits was much greater than that for qualitative traits, indicating the relative abundance of quantitative traits, and the mean Shannon–Weaver genetic diversity index values showed the order flower (1.53) > leaf (1.46) > fruit (1.40).

### 3.4. Correlations Between Phenotypic Traits

**Figure 3:**
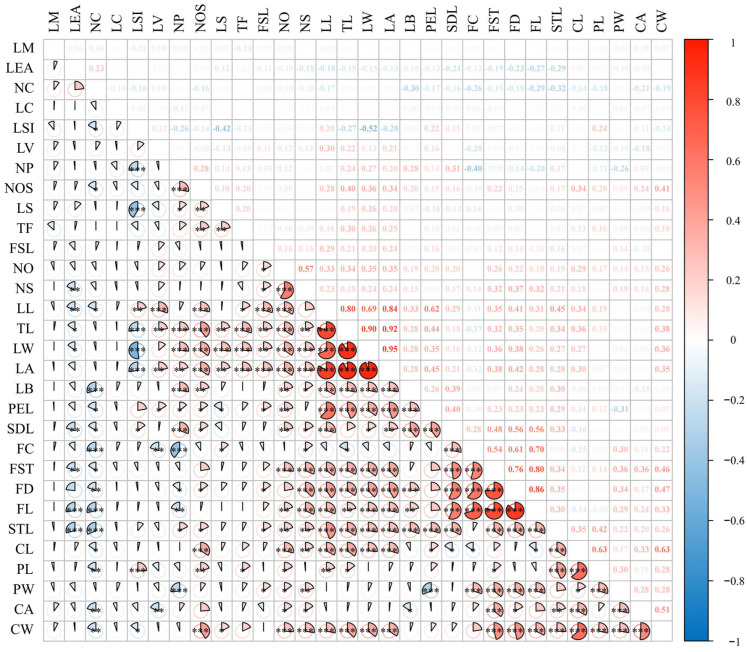
Correlation analysis of phenotypic traits of sect. *Tuberculate* plants. (* *p* < 0.1, ** *p* < 0.05, *** *p* < 0.01).

The results of the correlation analysis of 30 phenotypic traits from 18 populations of sect. *Tuberculate* plants indicated (Figure 3) that there were different degrees of correlation among the 30 phenotypic traits. Among them, there were 211 pairs of phenotypic traits with highly significant or significant correlations among different traits, 169 pairs with a positive correlations, and 42 pairs with negative correlations.

(1) There are 149 pairs of flowers and 12 pairs of FC that are negatively correlated with NC, LV, NP, TL, and CL and positively correlated with LS, NS, SDL, FST, FD, FL, and PW; 11 pairs of CA are negatively correlated with NC, LV, and LB and positively correlated with NO, NS, FST, FD, STL, CL, PW, and CW; 15 pairs of NC are negatively correlated with LSI, NOS, LL, LB, PEL, SDL, FC, FST, FD, FL, STL, CL, PL, CA, and CW; CL has 17 pairs that are negatively correlated with NC, SDL, FC, and FL and positively correlated with NOS, NO, NS, LL, TL, LW, LA, PEL, STL, PL, PW, CA, and CW. CW has 18 pairs that are negatively correlated with NC and LSI and positively correlated with NOS, LS, NO, NS, LL, TL, LW, LA, FST, FD, FL, STL, CL, PL, PW, and CA; NP has 14 pairs that are negatively correlated with LSI, FC, FD, FL, and PW and positively correlated with NOS, LS, TL, LW, LA, LB, PEL, SDL, and STL; PL has 12 pairs that are negatively correlated with NC and positively correlated with LSI, NOS, TF, NO, LL, TL, FST, STL, CL, PW, and CW. PW has 13 pairs that are negatively correlated with NP and PEL and positively correlated with FST, NO, NS, FC, FST, FD, FL, CL, PL, CA, and CW. STL has 21 pairs that are negatively correlated with LEA and NC and positively correlated with NP, NOS, FSL, NO, NS, LL, TL, LW, LA, LB, PEL, SDL, FST, FD, FL, CL, PL, CA, and CW; NOS has 16 pairs that are negatively correlated with NC and positively correlated with NP, LS, TF, LL, TL, LW, LA, LB, PEL, SDL, FD, STL, CL, PL, and CW. (2) There are 122 pairs of fruits; there are 7 pairs for TF that are positively correlated with NOS, LS, LL, TL, LW, LA, and PL; there are 18 pairs for FD that are negatively correlated with NC and NP and positively correlated with NOS, FSL, NS, LL, TL, LW, LA, LB, SDL, FC, FST, FL, STL, PW, CA, and CW; there are 10 pairs for FSL that are positively correlated with NO, NS, LL, TL, LW, LA, PEL, FD, STL, and PW; FL has 18 pairs that are negatively correlated with CL, LEA, NC, and NP and positively correlated with NO, NS, LL, TL, LW, LA, LB, SDL, FC, FST, FD, STL, PW, and CW; FST has 17 pairs that are negatively correlated with LEA and NC and positively correlated with NP, NOS, NO, NS, LL, TL, LW, LA, SDL, FC, FD, FL, STL, PL, PW, CA, and CW; SDL has 18 pairs that are negatively correlated with LEA, NC, and CL and positively correlated with LSI, NP, NOS, NO, NS, LL, LW, LA, LB, PEL, FC, FST, FD, FL, and STL; NO has 17 pairs that are positively correlated with FSL, NS, LL, TL, LW, LA, LB, PEL, SDL, FST, FL, STL, CL, PL, PW, CA, and CW; NS has 17 pairs that are negatively correlated with LEA and positively correlated with FSL, NO, TL, LW, LA, LB, SDL, FC, FST, FD, FL, STL, CL, PW, CA, and CW. (3) There are 149 pairs of leaves; there are 8 pairs for LEA, with NS, LL, TL, LW, SDL, FST, FL, and STL all being significantly or very significantly negatively correlated; there are 15 pairs for LB that are negatively correlated with NC and CA and positively correlated with NP, NOS, NO, NS, LL, TL, LW, LA, PEL, SDL, FD, FL, and STL; there are 6 pairs for LV that are positively correlated with LL, TL, LA, and PEL and negatively correlated with FC and CA; LSI has 9 pairs that are negatively correlated with NP, LS, TL, LW, LA, and CW and significantly positively correlated with LL, SDL, and PL; LA has 21 pairs that are negatively correlated with LSI, LV, NP, NOS, LS, TF, FSL, NO, NS, LL, TL, LW, LB, PEL, SDL, FST, FD, FL, STL, and positively correlated with CL and CW; LS has 9 pairs that are negatively correlated with LSI and PEL and positively correlated with NOS, TF, TL, LW, LA, FC, and CW; TL has 23 pairs that are negatively correlated with LSI, LEA, and FC and positively correlated with LV, NP, NOS, LS, TF, FST, NO, NS, LL, LW, LA, LB, PEL, FST, FD, FL, STL, SL, PL, and CW; PEL has 16 pairs that are negatively correlated with NC, LS, and PW and positively correlated with LV, NP, NOS, FSL, NO, LL, TL, LW, LA, LB, SDL, STL, and CL; LW has 21 pairs that are negatively correlated with LEA and LSI and positively correlated with NP, NOS, LS, TF, FSL, NO, NS, LL, TL, LA, LB, PEL, SDL, FST, FD, FL, STL, CL, and CW; LL has 21 pairs that are negatively correlated with LEA and NC and positively correlated with LSI, LV, NOS, TF, FSL, NO, TL, LW, LA, LB, PEL, SDL, FST, FD, FL, STL, CL, PL, and CW.

In summary, the STL, CW, and CL of flowers are correlated with several traits, the FD, FL, and SDL of fruits are correlated with several traits, the TL, LL, LW, and LA of leaves are correlated with several traits, and these can be used as an important index for screening good resources and classifying them.

### 3.5. Correlation Analysis of Phenotypic Traits with Geographical and Climatic Factors

**Figure 4:**
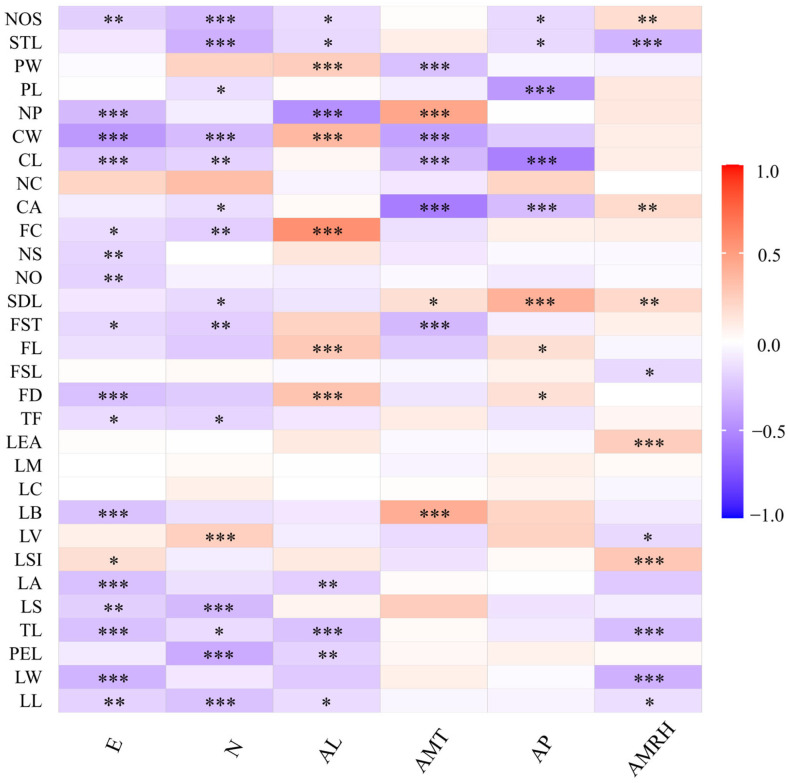
Correlation analysis of the 30 phenotypic traits and altitude and environmental factors. (* *p* < 0.1, ** *p* < 0.05, *** *p* < 0.01).

Thirty phenotypic traits were correlated with longitude, latitude, altitude, mean annual temperature, mean annual rainfall, and mean annual relative humidity. The results showed that some of the traits of sect. *Tuberculate* plants were significantly correlated with geographical and climatic factors (Figure 4). LSI shows a significant positive correlation with longitude (E), while NP, CW, CL, FD, LB, LA, TL, and LW show a highly significant negative correlation with longitude; LV shows a highly significant positive correlation with latitude (N), while NOS, STL, CW, LS, PEL, and LL show a highly significant negative correlation with latitude; PW, CW, FC, FL, and FD show a highly significant positive correlation with elevation; NP and TL show a highly significant negative correlation with altitude (AL); NP and LB show a highly significant positive correlation with annual mean temperature (AMT), while PW, CW, CL, and CA show a highly significant negative correlation with AMT; SDL shows a highly significant positive correlation with annual precipitation (AP), while PL, CL, and CA show a highly significant negative correlation with AP; LEA and LSI show a highly significant positive correlation with annual mean relative humidity (AMRH); and STL, TL, and LW show a highly significant negative correlation with AMRH. In summary, the phenotypic traits of flowers, fruits, and leaves of sect. *Tuberculate* plants were influenced to some extent by geographic and climatic factors, where AMRH had less effect on the phenotypic traits of flowers than the other factors, E, N, and AP had a greater effect on the phenotypic traits of fruits, and E, N, and AMRH had a greater effect on the phenotypic traits of leaves.

### 3.6. Principal Component Analysis of the Phenotypic Shape

**Figure 5:**
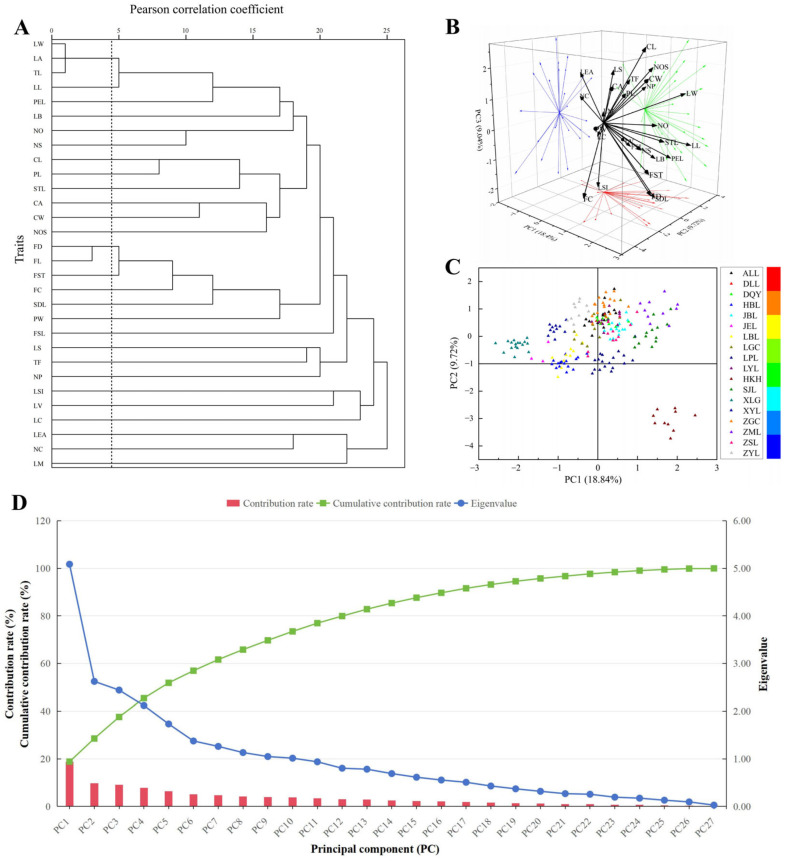
R-type clustering of phenotypic traits of sect. *Tuberculate* plants and principal component analysis. ((**A**): R-type clustering based on 30 phenotypic traits; (**B**): Three-dimensional projection of phenotypic traits on the first three principal components; (**C**): Scatterplot of the 212 samples constructed in terms of principal component 1 (PC1) and principal component 2 (PC2); (**D**): Eigenvalues, contributions, and cumulative contributions based on 27 phenotypic traits in the principal component analysis).

The results of an R-type cluster analysis of the 30 phenotypic traits from 18 populations of sect. *Tuberculate* plants showed (Figure 5A) that the degree of correlation among LW, LA, and TL was high and the difference among them not significant. The same was true for FD and FL, so one of these traits (LW and FD) was retained in each group. The other traits were not highly correlated with one another, so they could be used to fulfill different roles in the classification of the plants. Then, 27 phenotypic traits were selected for subsequent analyses. The results of the PCA of the 27 phenotypic traits screened through the screening process showed (Figure 5B,D and Table S2) that there were 10 principal components with eigenvalues greater than 1, which contributed from 3.76% to 18.84%, with a cumulative contribution of 73.49%.

(1) In principal component 1, the eigenvalue vectors of CW (0.29), STL (0.28), CL (0.21), and NOS (0.20) were larger in absolute value, mainly reflecting the width of the calyx and the length of the style; the eigenvalue vectors of FD (0.32), FST (0.32), SDL (0.23), NO (0.23), and NS (0.21) were larger in absolute value, mainly reflecting the diameter of the fruit and the thickness of the fruit shell; and LL (0.30) and LW (0.28) had larger absolute values of the eigenvalue vectors, mainly reflecting the width of the leaves. (2) In principal component 2, the eigenvalue vectors of FC (−0.43), PW (−0.36), NP (0.35), and CA (−0.23) had large absolute values, mainly reflecting the color of the flowers and the number and width of the petals; the eigenvalue vectors of FST (−0.22) had a large absolute value, mainly reflecting the thickness of the fruit shell; and the eigenvalues of PEL (0.32), LL (0.29), LW (0.26), LV (0.22), and LB (0.21) had larger absolute values of the eigenvalue vectors, mainly reflecting the length of the petiole. (3) In principal component 3, the larger absolute values of the eigenvalue vectors of CL (0.37), NOS (0.27), FC (−0.23), and CA (0.22) mainly reflected the length of calyx and the number of styles; the larger absolute values of the eigenvalue vectors of SDL (−0.32), FD (−0.22), and TF (0.21) mainly reflected the length of the seeds; and the larger absolute values of the eigenvalue vectors of LSI (−0.32), LS (0.25), and LEA (0.22) mainly reflected the leaf shape index. (4) In principal component 4, the absolute values of the eigenvalue vectors of PL (0.45), CL (0.35), and NP (−0.20) were larger, mainly reflecting the length of the calyx and petals; the absolute values of the eigenvalue vectors of FD (−0.22) were larger, mainly reflecting the diameter of the fruit; and the absolute values of the eigenvalue vectors of LSI (0.48), LS (−0.33), and LW (−0.23) were larger, mainly reflecting the shape characteristics of the leaves. (5) In principal component 5, the larger absolute values of the eigenvalue vectors of NC (0.34), NP (−0.28), and NOS (−0.21) mainly reflected the variation in the number of calyxes; the larger absolute values of the eigenvalue vectors of FSL (0.36), NS (0.33), NO (0.28), and SDL (−0.23) mainly reflected the length of the fruiting stalks and the number of seeds; and the larger absolute values of the eigenvalue vectors of LV (0.38) and LB (−0.27) mainly reflected the number of pairs of leaf veins and the type of leaf bases. (6) In principal component 6, the larger absolute values of the eigenvalue vectors of CA (−0.42) and NP (−0.32) mainly reflected the number of petals and the calyx apex; the larger absolute values of the eigenvalue vectors of TF (0.32), NS (−0.27), and NO (−0.21) mainly reflected the type of splitting of the fruit; and the larger absolute values of the eigenvalue vectors of LC (0.34), LS (0.33), LM (−0.23), and LL (0.22) mainly reflected the color of the leaves. (7) In principal component 7, the larger absolute values of the eigenvalue vectors of NC (0.25) and STL (−0.20) mainly reflected the number of calyxes; the larger absolute values of the eigenvalue vectors of NS (−0.33), NO (−0.31), and FST (0.23) mainly reflected the numbers of seeds and ovaries; and the larger absolute values of the eigenvalue vectors of LM (0.30), LEA (0.30), PEL (0.29), LB (−0.25), and LC (−0.22) mainly reflected the leaf margins and leaf apex. (8) In principal component 8, the larger absolute value of the eigenvalue vector of NC (−0.25) mainly reflected the number of calyxes; the larger absolute value of the eigenvalue vector of TF (−0.44) mainly reflected the type of splitting of the fruit; and the larger absolute value of the eigenvalue vectors of LM (0.59), LC (0.38), LS (0.23), and LB (0.22) mainly reflected the color of the leaf and the leaf margin. (9) In principal component 9, the absolute values of the eigenvalue vectors of STL (−0.33) and PW (−0.20) were larger, mainly reflecting the length of the style; the absolute values of the eigenvalue vectors of FSL (−0.46), NO (0.36), and NS (0.29) were larger, mainly reflecting the length of the fruit stalk; and the absolute values of the eigenvalue vectors of LEA (0.43) and LM (−0.30) were larger, mainly reflecting the leaf apex and leaf margin. (10) In principal component 10, the larger absolute values of the eigenvalue vectors of NC (0.37), PL (0.29), and CA (−0.28) mainly reflected the number of calyxes; the larger absolute values of the eigenvalue vectors of SDL (0.23) and FSL (0.22) mainly reflected the length of fruits and seeds; and the larger absolute values of eigenvalue vectors of LEA (0.44), LB (0.25), LM (0.24), LC (−0.22), and LV (−0.20) mainly reflected the leaf apex characteristics.

Scatterplots of the relationships among the samples were constructed using PC1 and PC2 as the horizontal and vertical coordinates, respectively (Figure 5C). The results showed that there were differences in phenotypic traits among the 18 populations of sect. *Tuberculate* plants, and the width of the calyx, length of the style, diameter of the fruit, thickness of the fruit shell, and narrowness of the leaves had a greater influence on the phenotypic traits of the plants of sect. *Tuberculate* than the color of the flowers, the number and width of petals, the thickness of the fruit shell, and the length of the petiole. The PC1 vector for HKH was large, and the PC2 vector was small, indicating that the populations as a whole presented morphological characteristics such as red flowers, larger fruit, and thicker shells. PC2 for XLG was larger than PC1, indicating that the populations as a whole presented morphological characteristics such as smaller leaves and petioles. The samples of the populations of HKH, XLG, *Camellia lipingensis* (LPL), *Camellia hupehensis* (HBL), and XYL were more concentrated, and the distribution points of the samples of the populations of ZGC, *Camellia anlungensis* (ALL), *Camellia obovatifolia* (DLL), *Camellia zengii* (ZSL), *Camellia acutiperulata* (JBL), and *Camellia ilicifolia* (DQY) overlapped more. The rest of the populations’ sample distributions were more scattered.

### 3.7. Cluster Analysis of Sect. Tuberculate Plants

**Figure 6:**
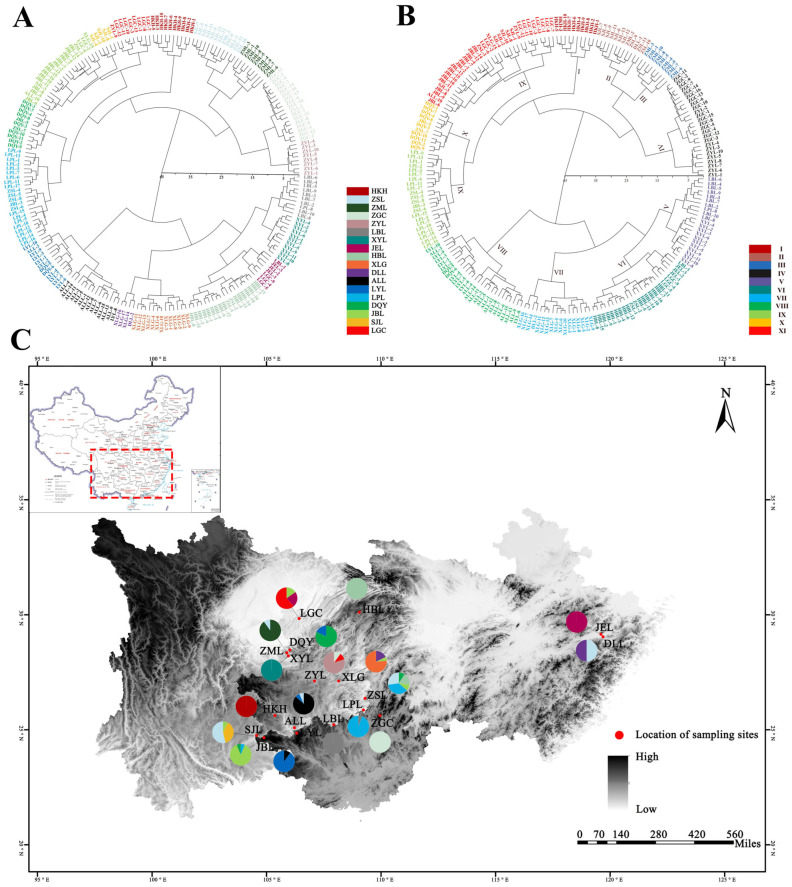
Q-cluster analysis of 212 samples of sect. *Tuberculate* plants. ((**A**): Cluster tree at a Euclidean distance of 7.5; (**B**): Distance tree at a Euclidean distance of 10; (**C**): Geographic distribution of 212 sect. *Tuberculate* plants in 18 populations).

In order to further investigate the affinities among the sect. *Tuberculate* plant populations, a systematic cluster analysis was carried out using Ward’s method based on the different expressions of 27 phenotypic traits among 18 sect. *Tuberculate* plant populations according to Euclidean distance. Dendrograms were used to visually reflect the proximity of the affinities between the taxonomic units. At a Euclidean distance of 7.5, the 212 sect. *Tuberculate* plants could be classified into 18 types (Figure 6A), suggesting that there were more distinctive differentiation features among the different populations. When the Euclidean distance was 10, the 212 sect. *Tuberculate* plants could be classified into 11 types (Figure 6B).

(1) Taxon I consists of one subclass with 10 samples from Zima Township, Qinglong County, Guizhou Province, and it is mainly characterized by reddish flowers, longer leaves, thicker fruit shells, and larger fruit diameters. (2) Taxon II consists of three subclasses with a total of 12 specimens, mostly from Sanjiangkou Town, Xingyi City, Guizhou Province, and Wulong Mountain, Qutuan Village, Liping County, Guizhou Province, and its main characteristics are white flowers, rounded sepal apices, and oval or long oval leaves. (3) Taxon III consists of one subclass with a total of nine specimens from Dawuji, Yuanhou Town, Chishui City, Guizhou Province, and it is mainly characterized by a pointed sepal apex, oval or long oval leaves, and serrated leaf margins. (4) Taxon IV consists of two subclasses with a total of 25 specimens from Laomao, Tianping Mountain, Longsheng Autonomous County, Guangxi Province, and Baixianglin, Shuangshan Village, Fengsan Township, Kaiyang County, Guizhou Province, and it is mainly characterized by sepals that are rounded at the apex, three or four styles, lanceolate leaves with serrated margins, and white flowers. (5) Taxon V consists of two subclasses with a total of 21 specimens from Moganshai, Libo County, Guizhou Province, and Hutou Mountain, Yuanhou Town, Chishui City, Guizhou Province, and it is mainly characterized by rounded sepal apices, long oval or lanceolate leaves, and longer petioles. (6) Taxon VI consists of five subclasses with a total of 25 specimens, most of which are mainly from Maoba Township, Lichuan City, Hubei Province, and it is mainly characterized by rounded sepal apices, oval or lanceolate leaves, and longer petals. (7) Taxon VII consists of two subclasses with a total of 17 specimens, most of which are from Yuntai Mountain, Shibing County, Guizhou Province, and it is mainly characterized by rounded sepal apices, oval leaves, smaller leaves, short petioles, white flowers, and leaf margins with microwaveable serrations. (8) Taxon VIII comprises three subclasses with 26 specimens, most of which are mainly from Liaokang Village, Zhexiang Town, Wangmo County, Guizhou Province, and Xiangjiaping, Yachang Town, Leye County, Guangxi Province, and it is characterized by rounded sepals at the apex, oval leaves, white flowers, and a large number of petals. (9) Taxon IX consists of three subclasses with a total of 22 specimens, most of which are mainly from beside barren fields in Liping County, Guizhou Province, and it is mainly characterized by pointed sepal apices, lanceolate leaves, and white flowers. (10) Taxon X consists of three subclasses with a total of 11 specimens, most of which are mainly from Jinshagou, Chishui City, Guizhou Province, and it is mainly characterized by rounded sepal apices, serrated leaf margins, longer petioles, and white flowers. (11) Taxon XI consists of seven subclasses with a total of 34 samples, most of which are mainly from Jinzhongshan Township, Longlin County, Guangxi Province, Wild Monkey Valley, Sanjiangkou Township, Xingyi City, Guizhou Province, and Miaomian, Jinyun Mountain, Chongqing Municipality, and it is mainly characterized by pointed sepal apices, oval leaves, and white flowers.

At the same time, the geographical distribution of the 18 populations was projected based on the results of the clustering analysis of sect. *Tuberculate* plants (Figure 6C). The results showed that the 212 plant samples were not fully clustered according to their geographical distance, and there was overlap between some populations.

## 4. Discussion

Phenotypic diversity analysis is an important tool for studying the variations in morphological, physiological, and biochemical characteristics exhibited by organisms under different environmental conditions. In the case of woody plants, a variety of factors, such as the environment in which a species is located, its ecological niche, and its interactions with other species, can lead to morphological, structural, and functional variation by influencing the growth, development, and physiological processes of the plants [40]. The plants of sect. *Tuberculate* spread from Guizhou as the center of their distribution to neighboring provinces, and the environmental factors in the areas in which they are distributed vary greatly [22]. In addition to directly affecting phenotypic variation, these environmental factors can indirectly influence the phenotypic diversity of woody plants by affecting their genetic differentiation. When plants are distributed in different environments, as a result of natural selection, some genotypes that are able to adapt to specific environments may be retained and gradually dominate in the population. Over time, these adaptive genotypic differences may accumulate among populations, leading to genetic differentiation and further exacerbating the degree of phenotypic variation among plants in different environments.

Phenotypic trait variation plays an important role in plants’ adaptation to different habitats and evolution [1,2]. The phenotypic coefficient of variation can reflect the potential of plants to adapt to the environment, and a higher coefficient of variation indicates a greater ability of plants to adapt to the environment [41]. In this study, the diversity indices of 30 phenotypic traits of sect. *Tuberculate* plants ranged from 0.48 to 2.07, with a mean value of 1.47, and 21 traits had a diversity index greater than 1, which indicated that the phenotypic traits of the sect. *Tuberculate* plants had a high degree of homogeneity and richness. The coefficients of variation of the 21 quantitative traits of the sect. *Tuberculate* plants were different, and the differences in the magnitude of variation of each trait were obvious, indicating that there were differences in the adaptation of different traits to the environmental changes: the mean value of the coefficient of variation (CV) was 14.88%, and the largest one was 27.98%, which indicated that the diversity of the species was high, and it possessed a strong ability to adapt to the environment. It was further found that the sect. *Tuberculate* plants differed greatly in traits among populations, while most of the traits did not differ much within populations: the mean value of the phenotypic differentiation coefficient of the 30 phenotypic traits of the sect. *Tuberculate* plants was 61.405, and the mean value of the variance components within populations (32.705%) was smaller than the mean value of the variance components among populations (47.363%), indicating that the differentiation of the phenotypic traits of sect. *Tuberculate* plants existed both within populations and among populations, but mainly among populations. We hypothesize that this phenomenon is mainly caused by differences in climatic and environmental conditions and that the geographically discontinuous distribution of most populations greatly contributes to the growth and development of species and the exchange of genes between their populations [42]. The mean value of the phenotypic diversity index of the 30 phenotypic traits of the sect. *Tuberculate* plants was 1.3, which was significantly higher than that of *Camellia oleifera* (1.06) [43] and sect. *Thea* (0.91) [44], indicating that sect. *Tuberculate* plants had rich phenotypic diversity. Moreover, there was no significant correlation between the diversity indices and coefficients of variation for the 30 phenotypic traits in the sect. *Tuberculate* plants, which was the same as the findings for the other plants [45]. Overall, the magnitude of phenotypic variation was greater for fruits than for leaves, suggesting that the reproductive organs of plants in this taxon may have greater phenotypic plasticity in the face of environmental stress, making it easy for their populations to reproduce.

Plant traits are not isolated from each other, but adapt to changes in the external environment by forming synergistic or replicative trade-off relationships with one another [46]. In this study, the NP was negatively correlated with petal size (PL and PW), suggesting that there is a trade-off relationship where one part decreases and the other part increases in the allocation of reproductive resources in sect. *Tuberculate* plants. This is consistent with the hypothesis of a trade-off between the flower number and size in plant reproductive allocation theory [47,48]. Secondly, the leaf area was highly significantly and positively correlated with the petal number and fruit size and negatively correlated with the flower size (petal length and width), indicating that the larger the leaf area of plants in sect. *Tuberculate*, the greater the number of petals, the larger the fruit, and the smaller the flower morphology. This may be due to the fact that a larger leaf area can retain more water, which is conducive to promoting the formation of flowers and fruit organs, increasing the number of petals and fruit size; however, it is difficult for plants to distribute their resources evenly, so they adopt a trade-off strategy between the number of petals and the flower size. At the same time, there was a significant correlation between leaf phenotypic traits and some of the floral phenotypic traits, suggesting that there was an interdependence between reproductive organs and nutrient organs in sect. *Tuberculate* plants. The phenotypic traits of flowers, fruits, and leaves of sect. *Tuberculate* plants showed significant correlations with one another overall, suggesting a complex and close relationship among the phenotypic traits, similar to that in *Camellia* L., such as *Camellia oleifera* and sect. *Thea* [43,44]. However, for the adaptive mechanisms among these traits, further exploration in the direction of ecological mechanisms and molecular biology may be required. It has been shown that the phenotypic traits of a wide range of woody plants are significantly influenced by geo-climatic factors and that the degree of adaptation and sensitivity to the environment in which they are found varies from plant to plant, resulting in different patterns of geographic variation [49]. For example, *Camellia drupifera* seed traits are highly correlated with factors such as latitude, longitude, and altitude [50]. The results of the present study showed that the phenotypic traits of sect. *Tuberculate* plants were significantly affected by longitude, latitude, and altitude. The number of petals, calyx size, and fruit size decreased with increasing longitude. The number of leaf vein pairs increased the style length, calyx width, leaf morphology, petiole length, leaf size, and number of styles decreased progressively with increasing latitude, and the fruit size and number of petals increased and number of petals and leaf types decreased with increasing altitude. This coincides with the distribution of the genus *Camellia*, which is mainly in the middle and lower reaches of the Yangtze River and the southern regions of China, with a preference for acidic soils and a particular preference for warm and humid environments [21]. Meanwhile, the increase in AP and AMRH was accompanied by an increase in the length of seeds and a decrease in the length of petals and the calyx. This indicates that different plants have different adaptability to different environments, thus producing different variation patterns. Therefore, we should fully consider the adaptability of the characteristics of good seeds to environmental factors during the promotion of good species of sect. *Tuberculate* plants.

R-type clustering is a classification of traits that can reflect the correlations between traits and the reasonableness of their selection [51]. James first used R-type clustering in quantitative classification, and then it was widely used in woody plants [52]. In the present study, 30 phenotypic traits were selected to differentiate sect. *Tuberculate* plants, and based on the results of R-type clustering and correlation analysis, LA, TL, and FL were deleted from sect. *Tuberculate* plants and more representative traits (LW and FD) were selected. Therefore, 27 more desirable phenotypic traits that can be used as taxonomic indices for sect. *Tuberculate* plants were finally selected. Principal component analysis can be used to screen the core indicators from a large number of trait indicators to maximally represent the original data [39]. In this study, 10 principal components with eigenvalues greater than 1 were extracted for 27 phenotypic traits, with a cumulative contribution of 73.49%, which reflected most of the information on the phenotypic traits. The principal component vector scatterplots distinguished some populations of sect. *Tuberculate* (HKH, XLG); however, most of the populations were relatively concentrated and partially overlapped, which also indicated the overlapping of traits and the difficulty of the taxonomic identification of this taxon. Meanwhile, 12 traits of leaf length, leaf width, leaf color, leaf apex, fruit diameter, fruit shell thickness, flower color, calyx apex, calyx width, petal length, petal width, and the number of styles were screened using a combination of principal component analyses and correlation analyses, and these were then used as the main traits for distinguishing and evaluating sect. *Tuberculate* plants.

In traditional classification, this taxon is subject to considerable taxonomic controversy, with Chang and Min et al. classifying it into 18 and 6 species based on different taxonomic views [16,17,18,19,20,21]. The results of Q-clustering analysis based on phenotypic traits in this study showed that 212 sect. *Tuberculate* plants were divided into 18 groups when the genetic distance was equal to 7.5, indicating that this study supported the classification of sect. *Tuberculate* plants into 18 species. When the genetic distance was equal to 10, the 212 sect. *Tuberculate* plants were classified into 11 major groups, and this clustering result had both similarities and differences in comparison with the traditional classification. For example, the clustering results of groups V and VIII illustrated the existence of similar phenotypic traits between LBL and XYL and between ALL and LYL, which made it more difficult to differentiate between them. At the same time, it further supported the finding that XYL and DQY are in separate species [28]. The clustering results for group I indicate that this group of plants, with its larger flower diameter, denser branches and leaves, larger fruits, and brighter and more distinctive flower colors, is an important parent for the selection of good new varieties, with high ornamental value and potential medicinal value, and it has a wide range of prospects for promotion. The cluster grouping results for the 18 populations of sect. *Tuberculate* plants were closely related to flower color, petal size, number of petals, calyx apex, style length, fruit shell thickness, fruit diameter, leaf size, leaf margin, leaf shape, and petiole length. The clustering results can provide basic data for the cross-breeding and phenotypic diversity of hybrids of sect. *Tuberculate* plants. Overall, the phenotypic characteristics of the 18 populations of sect. *Tuberculate* were not completely clustered according to their geographic distribution, indicating that geographic isolation is not a key factor in the phenotypic differentiation of sect. *Tuberculate* populations. This may be due to the unique geography of the karst region and its climatic factors, where populations are in different microclimates, leading to their phenotypic changes in response to climate change [53]. During the field survey, we found that three populations, XYL, DQY, and *Camellia atuberculata* (ZML), existed in the Yuanhou Town area of Chishui City, Guizhou Province, with their main distributions being dominated by shrubs, slopes, and valleys, and the unique microclimate formed in the area made the phenotypic diversity of the populations unusually complex. Some populations distinct from the three taxa were found, and we hypothesized that they might be a transitional population. Based on these phenotypic traits, future research can be carried out to evaluate the resources of sect. *Tuberculate* plants, select and breed good seeds and identify them, and further explore the genetic structure and evolutionary history of plant populations by combining these methods with genetic methods (e.g., molecular marker analysis, genome sequencing). At the same time, the maximum entropy model and other means can be used to predict the geographic distribution of plants and explore the relationship between competition and coexistence so as to provide a solid basis for the conservation of germplasm resources of the tea group of plants and the creation of new germplasms in a highly efficient manner. We will also use the maximum entropy model and other means to predict the geographical distribution of plants and explore the relationship between species competition and coexistence so as to provide a solid foundation for the conservation of germplasm resources and the efficient creation of new germplasms of sect. *Tuberculate* plants.

## 5. Conclusions

Sect. *Tuberculate* plants are rich in phenotypic variation, with large differences in the magnitude of variation for different phenotypic traits, and the variation mainly occurs among populations. Among the different populations, the magnitude of variation was highest in the SJL population and smallest in the ZGC population. The coefficient of variation between different traits was largest in FSL and smallest in NP (6.66%), which was relatively stable, and the mean values of the coefficients of variation were in the order fruit (18.57%) > leaf (17.85%) > flower (9.41%). The sect. *Tuberculate* plants had rich phenotypic diversity, with little difference in diversity indices among phenotypes, and the phenotypic diversity indices for quantitative traits were much greater than those for qualitative traits (flowers (1.53) > leaves (1.46) > fruits (1.40)). There are complex and close relationships between the various phenotypic traits, while flowers, fruits, and leaves differ in their adaptive choices according to geographic and climatic factors. The principal component analysis of 27 major traits screened using R-type cluster analysis showed that the leaf length, leaf width, leaf color, leaf apex, fruit diameter, fruit shell thickness, flower color, calyx apex, calyx width, petal length, petal width, and number of styles were the major traits contributing to the phenotypic differences in sect. *Tuberculate*. A Q-type analysis based on phenotypic traits showed that the clustering results formed at Euclidean distances of 7.5 and 10 were somewhat different from the traditional classification. This study analyzed the diversity of phenotypic traits in sect. *Tuberculate*, and the results will provide a scientific basis for genetic diversity, resource identification, interspecific classification, and the development and utilization of germplasm resources.

## Figures and Tables

**Table 1:** Geographic, climatic, and sampling information of the 18 populations of sect. *Tuberculate*.

Population	Sample Size	Altitude (m, AL)	Longitude (°E, E)	Latitude (°N, N)	Annual Mean Temperature (°C, AMT)	Annual Precipitation (mm, AP)	Annual Mean Relative Humidity (%, AMRH)
*Camellia anlungensis* Chang (ALL)	15	418	106.13	25.20	19.2	1223	75
*Camellia obovatifolia* Chang (DLL)	2	35	119.35	29.70	17.9	1546	73
*Camellia ilicifolia* Y.K.Li (DQY)	11	730	106.00	28.27	18.6	1293	84
*Camellia hupehensis* Chang (HBL)	16	833	109.10	30.40	16.5	1452	82
*Camellia acutiperulata* Chang and Ye (JBL)	16	855	104.52	24.39	19.8	1124	71
*Camellia acutisepala* Chang (JEL)	3	35	119.35	29.70	17.9	1546	73
*Camellia rubimuricata* Chang and Z.R.Xu (LBL)	10	822	107.55	25.13	18.8	1345	79
*Camellia tuberculata* Chien (LGC)	13	696	106.23	29.50	17.6	1437	40
*Camellia lipingensis* Chang (LPL)	18	743	109.14	26.40	16.2	960	76
*Camellia leyeensis* Chang and Y.C.Zhong (LYL)	10	656	106.17	24.50	17.1	1266	83
*Camellia rubituberculata* Chang and Yu (HKH)	10	1394	105.19	25.39	16.4	1312	78
*Camellia pyxidiacea* Xu F.P.Chen and C.Y.Deng (SJL)	13	931	104.32	24.47	16.4	1312	78
*Camellia parvimuricata* Chang (XLG)	17	886	108.60	27.60	17.9	1251	79
*Camellia neriifolia* Hung T.Chang (XYL)	11	982	105.56	28.20	18.6	1293	82
*Camellia rhytidocarpa* Chang and Liang (ZGC)	16	659	109.54	25.38	18.1	1613	73
*Camellia atuberculata* Chang (ZML)	10	382	105.55	28.20	18.6	1293	82
*Camellia zengii* Chang (ZSL)	11	647	109.16	26.21	16.2	960	76
*Camellia rhytidophylla* Y. K. Li and M.Z.Yang (ZYL)	10	731	107.40	27.60	16.7	1168	74

**Table 2:** Quality traits of sect. *Tuberculate* plants and their abbreviations.

No.	Character	Abbreviation	Grading Assignment			
			1	2	3	4
1	Type of leaf	TL	Small leaves	Medium-sized leaves	Large leaves	Extra-large leaves
2	Leaf shape	LS	Nearly round	Lanceolate	Oval	Long oval shape
3	Leaf bases	LB	Rotundity	Blunt	Wedge	
4	Leaf color	LC	Light green	Green	Yellowish green	Dark green
5	Leaf margin	LM	Microwaveable	Undulate	Sessile	
6	Leaf apex	LEA	Acute	Papery	Blunt	
7	Type of splitting fruit	TF	Vertical fracture	Split open	Reticulated fissure	
8	Flower color	FC	White	Pink	Red	
9	Calyx apex	CA	Apex	Apex rounding		

**Table 3:** Quantitative traits of sect. *Tuberculate* plants and their abbreviations.

No.	Character	Abbreviation	Unit
1	Leaf length	LL	cm
2	Leaf width	LW	cm
3	Petiole length	PEL	cm
4	Leaf area	LA	cm^2^
5	Leaf shape index	LSI	-
6	Leaf veins	LV	-
7	Fruit diameter	FD	cm
8	Fruit stalk length	FSL	cm
9	Fruit length	FL	cm
10	Fruit shell thickness	FST	cm
11	Seed length	SDL	cm
12	Number of ovary	NO	-
13	Number of seeds	NS	-
14	Number of calyxes	NC	-
15	Calyx length	CL	cm
16	Calyx width	CW	cm
17	Number of petals	NP	-
18	Petal length	PL	cm
19	Petal width	PW	cm
20	Style length	STL	cm
21	Number of styles	NOS	-

**Table 4:** Variance components of phenotypic traits and phenotypic differentiation coefficients of different populations of sect. *Tuberculate* plants.

Phenotypic Trait	Among Populations		Within Populations		Random Error	Phenotypic Differentiation Coefficients (%)
	*F* Value	Variance Component/%	*F* Value	Variance Component/%	Variance Component/%	
LL	13.215 **	46.915	10.541 *	41.876	11.209	52.837
LW	2.711 **	46.444	1.947	43.372	10.183	51.710
PEL	0.224 *	49.658	0.184	28.504	21.838	63.532
TL	1.205	45.775	1.119 *	41.887	12.339	52.218
LS	1.105 *	48.417	0.425	21.595	29.988	69.155
LA	41.248 *	46.380	35.146 **	41.055	12.565	53.045
LSI	0.975 **	48.919	0.581	33.468	17.613	59.378
LV	3.534	47.970	1.648 *	24.051	27.980	66.606
LB	0.682 *	48.954	0.266	25.894	25.152	65.405
LC	1.784	47.841	1.294 *	3.483	48.676	93.214
LM	0.138 *	48.148	0.125	4.621	47.231	91.243
LEA	0.416 *	48.683	0.028	9.760	41.557	83.300
TF	0.342	47.580	0.047 *	11.769	40.651	80.170
FD	1.653 **	46.931	1.528	48.950	4.119	48.947
FSL	0.144	46.705	0.026	13.700	39.595	77.320
FL	1.238 **	46.557	1.059 *	48.791	4.651	48.829
FST	0.264 *	46.280	0.241	47.837	5.882	49.173
SDL	0.359 *	49.079	0.305	39.962	10.959	55.119
NO	0.451	48.587	0.286	14.713	36.700	76.757
NS	0.982	47.796	0.751 *	13.989	38.215	77.359
FC	0.326 **	41.170	0.311	50.266	8.564	45.026
CA	0.427 *	48.690	0.357 *	43.797	7.513	52.645
NC	2.756 **	50.277	1.974	30.238	19.485	62.444
CL	0.157 *	48.983	0.141 *	45.152	5.865	52.035
CW	0.054	48.479	0.024	46.054	5.467	51.283
NP	5.716	47.405	5.945	47.464	5.131	49.969
PL	1.024 *	50.661	0.812 *	41.018	8.320	55.259
PW	0.124	49.112	0.081	43.138	7.750	53.238
STL	0.354 *	41.152	0.214	36.324	22.524	53.116
NOS	0.229	41.337	0.218	38.437	20.226	51.818
Mean	2.795	47.363	2.254	32.705	19.932	61.405

Note: * *p* < 0.05 and ** *p* < 0.01.

**Table 5:** Frequency and diversity indices for each classification of quality traits.

Traits	Distribution Frequency of Each Grade				Diversity
	1	2	3	4	
TL	58	93	38	23	1.27
LS	9	108	55	40	1.14
LB	6	72	134	-	0.76
LC	47	76	27	62	1.32
LM	23	182	7	-	0.48
LEA	80	103	29	-	0.99
TF	173	29	10	-	0.58
FC	192	10	10	-	0.68
CA	110	102	-	-	0.69

## Data Availability

Data are contained within the article.

## Supplementary Materials

The following supporting information can be downloaded at https://www.mdpi.com/article/10.3390/plants13223210/s1. Table S1: Diversity of 21 quantitative traits in 18 populations of sect. *Tuberculate* plants; Table S2: Eigenvector values, eigenvalues, and cumulative contributions of each principal component of phenotypic traits of 212 sect. *Tuberculate* plants.
